# Size-induced twinning in InSb semiconductor during room temperature deformation

**DOI:** 10.1038/s41598-021-98492-w

**Published:** 2021-10-01

**Authors:** Florent Mignerot, Bouzid Kedjar, Hadi Bahsoun, Ludovic Thilly

**Affiliations:** Institut Pprime, Université de Poitiers – CNRS – ENSMA, SP2MI, Futuroscope, 86 962 Poitiers, France

**Keywords:** Semiconductors, Materials science, Condensed-matter physics

## Abstract

Room-temperature deformation mechanism of InSb micro-pillars has been investigated via a multi-scale experimental approach, where micro-pillars of 2 µm and 5 µm in diameter were first fabricated by focused ion beam (FIB) milling and in situ deformed in the FIB-SEM by micro-compression using a nano-indenter equipped with a flat tip. Strain rate jumps have been performed to determine the strain rate sensitivity coefficient and the related activation volume. The activation volume is found to be of the order of 3–5 b^3^, considering that plasticity is mediated by Shockley partial dislocations. Transmission electron microscopy (TEM) thin foils were extracted from deformed micro-pillars via the FIB lift-out technique: TEM analysis reveals the presence of nano-twins as major mechanism of plastic deformation, involving Shockley partial dislocations. The presence of twins was never reported in previous studies on the plasticity of bulk InSb: this deformation mechanism is discussed in the context of the plasticity of small-scale samples.

## Introduction

Semiconductors are known to be brittle at room temperature (and room pressure) but at elevated temperature, a transition occurs where the material presents a ductile behavior. This brittle-to-ductile transition (BDT) is generally situated around $0.6\times{\text{T}}_{\text{m}}$ where ${\text{T}}_{\text{m}}$ is the melting point of the material. Since 1950s, numerous studies were conducted to understand plasticity mechanisms in the different semiconductor materials and it is now widely accepted that the occurrence of the BDT is associated to a change in the nature of the dislocations^1,2^. Traditionally, investigation of the plasticity of brittle materials, in the form of bulk single crystals or polycrystals, requires the use of a deformation technique imposing a hydrostatic pressure component to prevent/delay fracture to the benefit of plasticity^3–6^. In such conditions favorable to plasticity, it has been shown that the Shuffle–Glide transition plays a major role in the BDT of semiconductors. Indeed, two gliding modes are possible in FCC or zinc-blende semiconductors with respect to the motion of dislocations in $\langle {111}\rangle$ planes: the Shuffle Set (SS) formed by two widely spaced $\langle {111}\rangle$ planes and the Glide Set (GS) formed by two closely spaced $\langle {111}\rangle$ planes. The nature of dislocations in the SS and the GS is in general different and Shockley partial dislocations can only exist in the GS due to geometrical considerations^7,8^.

The BDT has been studied in bulk Indium Antimonide, InSb, single crystals: first, the BDT occurs at T = 150 °C and, second, two transitions were evidenced with temperature^5,6^. In the ductile regime, above T = 150 °C, dissociated perfect dislocations are observed in the GS by transmission electron microscopy (TEM). Between 20 and 150 °C, i.e., in the brittle regime, plastic deformation takes place via the motion of only leading Shockley partial dislocations in the GS and/or widely dissociated perfect dislocations, also in the GS. At 20 °C, there is a coexistence of partial dislocations and non-dissociated perfect dislocations: it was suggested that these non-dissociated dislocations could belong to the SS. Below 20 °C and down to − 176 °C, only non-dissociated perfect dislocations are observed, as a further suspicion of a transition to the SS in a domain characterized by low temperature and high stress^6^. Hence, it was concluded that bulk deformation of InSb single crystal exhibits two transitions in temperature: the first one at room temperature associated to a change from SS to GS modes (characterized by a transition from non-dissociated perfect dislocations to partial dislocations) and the second one at about 150 °C (corresponding to the classical BDT) associated to a change from partial to perfect dislocations, both in the GS.

Very recently, Kumar et al.^9^ determined the generalized stacking fault energy (GSFE) by ab initio simulation (Density Functional Theory) and molecular dynamic (MD) for InSb and considered both SS and GS. GSFE results confirmed that the dislocations can easily dissociate in the GS while the dissociation is not energetically favorable in SS. In addition, atomistic calculations enabled to compare the ideal shear strength associated to the activation of both SS and GS: it was confirmed that, at low temperature, the motion of perfect dislocations in the SS is dominant, while as the temperature increases, dissociated dislocations in the GS becomes favorable. These atomistic calculations thus confirmed the conclusions drawn from previous experimental results in InSb bulk samples.

In the late 2000s, it was shown that the creation of cracks can be suppressed in (some) semiconductors by reducing the size of the samples^10,11^. Studies suggested different hypotheses to explain this so-called “size effect”: the reduced presence of native defects in small volumes^12^, the increase of the fracture energy^13^ or the drop of the crack propagation driving force^11^.

The fabrication of small-size samples can be obtained by different techniques, including ones derived from lithography. One of the most versatile techniques is based on milling by *focused ion beam* (FIB). However, one of the main drawbacks of FIB milling is associated to the production of damage in the crystal lattice that may lead to the formation of an amorphous layer at the sample surface^14,15^ or ion implantation and subsequent formation of lattice defects (vacancies, dislocations) in the sample volume. Most materials are suffering from this drawback, including semiconductors such as silicon and III-V compounds^16–18^. Fortunately, it was also shown in some studies that reducing the FIB current/voltage, milling time or beam angle with respect to the sample surface may limit drastically the impact of the ion beam on the crystal structure. In other words, an in-depth analysis of operational conditions of the FIB may allow defining parameters for which FIB-induced damage can be controlled if not avoided.

Size dependency on InSb micro-pillars have been studied by Thilly et al*.*^19^ to characterize the deformed microstructure and identify the plasticity mechanisms. An increase in the yield strength was evidenced on the mechanical curves with a reduction of micro-pillar size (diameter and height). Stacking faults were observed on a TEM lamella of a deformed InSb micro-pillar, suggesting that plastic deformation proceeds by the nucleation and glide of Shockley partial dislocations. More recently, size effect combined with the temperature dependency have been studied by Wheeler et al.^20^ on InSb micro-pillars. Strain rate jumps were performed to calculate the apparent activation volume with respect to the temperature. Nucleation and glide of Shockley partial dislocation was proposed to be the favorable mechanism at room temperature with an activation volume of $4.4 \pm 0.5 \,{\text{b}}^{3}$.

In the present work, the deformation of InSb micro-pillars of 2 µm and 5 µm in diameter is studied using in situ micro-compressions (constant strain rate tests and strain rate jumps tests) which allow for the determination of activation volumes. TEM and high-resolution TEM (HRTEM) analyses are then presented to characterize the deformed microstructure and plasticity mechanisms are identified. Finally, results are compared with the literature and are discussed to gain insight into the plasticity of InSb micro-pillars at room temperature using micro-compression.

## Results

**Figure 1:**
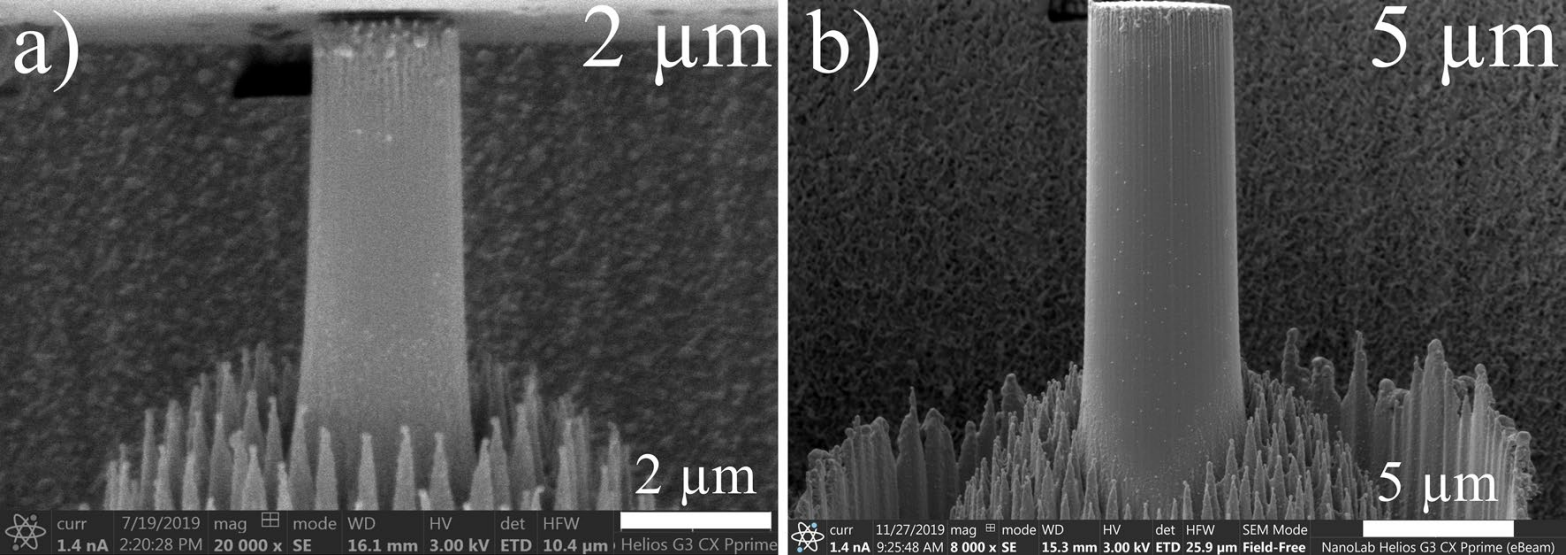
InSb micro-pillars before deformation taken with an observation angle (tilt) of − 2°.

Figure 1 shows two SEM images of InSb micro-pillars before deformation which exhibit a cylindrical shape with an aspect ratio of about 3 and a taper of about 3.5°. It can be noticed the presence of particles at the top surface due to the redeposition occurring at the height adjustment final step. A slight degradation of the top of the micro-pillars is visible on the SEM images which is directly also caused by this final step. These particles are amorphous and have no cohesion with the micro-pillar^19^: they have therefore no effect on the (plastic) deformation of the micro-pillars’ body. Some shallow stripes are visible along the micro-pillars surface: they are caused by the milling process (so-called “curtaining”). Columnar structures all around the pillars result from the chemical reaction between Ga and In atoms (see Methods section for details).

**Figure 2:**
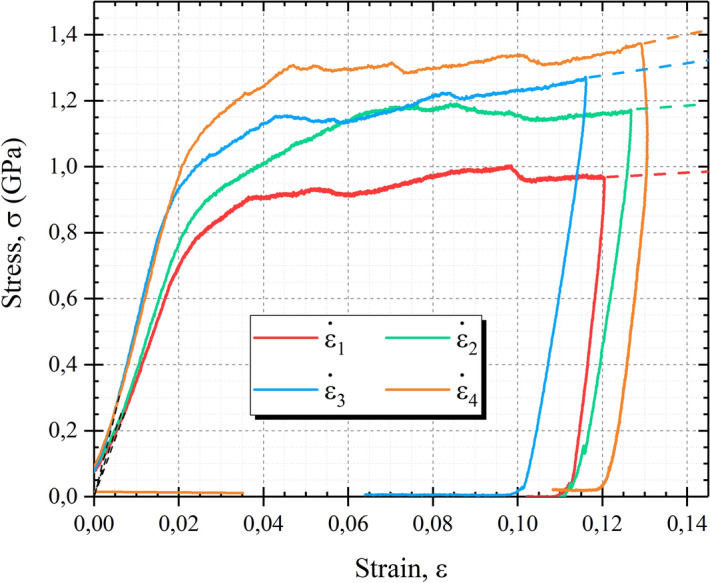
Stress–strain curves of InSb micro-pillars of 5 µm deformed by micro-compression with four constant strain rates (${\dot{\varepsilon}}_{1} = 1.5\times {10}^{-4} \, {\text{s}}^{-1}$, ${\dot{\varepsilon}}_{2} = 5\times {10}^{-4} \, {\text{s}}^{-1}$, ${\dot{\varepsilon}}_{3} = 1.0\times {10}^{-3} \, {\text{s}}^{-1}$, ${\dot{\varepsilon}}_{4} = 2\times {10}^{-3} \, {\text{s}}^{-1}$).

Figure 2 presents four stress–strain curves collected during the in-situ compression tests of InSb micro-pillars with different constant strain rates. Raw curves exhibit an initial non-linear segment associated to the small misalignment between the flat tip and the top of the micro-pillar. This non-linear segment has been removed by shifting the raw curves until the elastic part coincides with origin of the graph. The misalignment is also responsible for the variation of the elastic slope which should normally be identical for all micro-pillars with similar crystallographic orientation. Unloading slopes appear much more similar in the different curves, which is consistent with an identical crystal orientation. The curves present an extended plastic domain with several plastic events characterized by small stress drops. Stress hardening is also visible between these stress drops and it increases with increasing the strain rate as shown on Fig. 2 by comparing the slope at the end of the curves (dashed lines). Yield stress increases with the strain rate and similar trends were obtained with 2 µm micro-pillar series.

**Figure 3:**
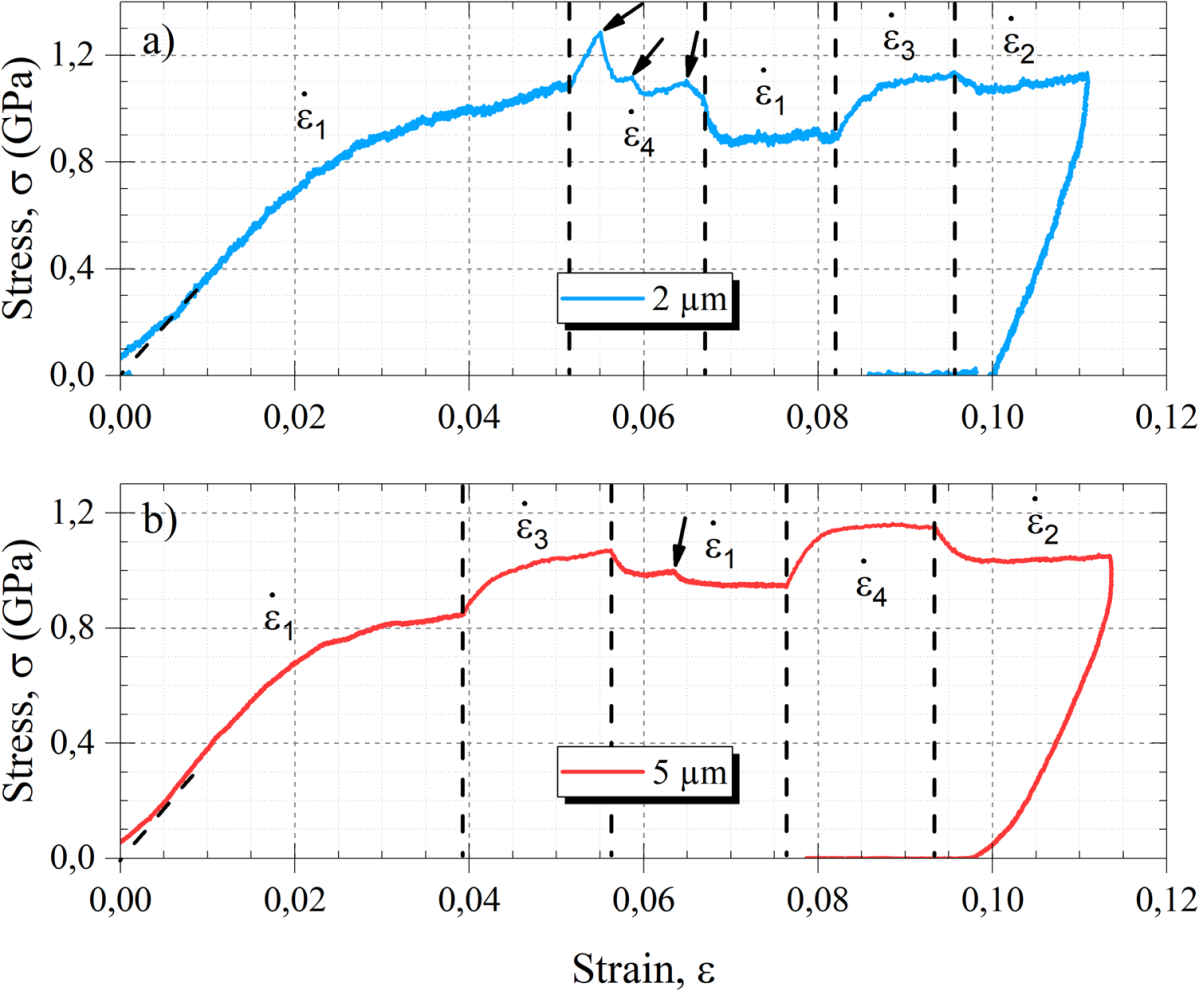
Stress–strain curves of InSb micro-pillars deformed with the strain rate jumps in (**a**) a diameter of 2 µm and in (**b**) 5 µm (${\dot{\varepsilon}}_{1} = 1.5\times {10}^{-4} \, {\text{s}}^{-1}$, ${\dot{\varepsilon}}_{2} = 5\times {10}^{-4} \, {\text{s}}^{-1}$, ${\dot{\varepsilon}}_{3} = 1.0\times {10}^{-3} \, {\text{s}}^{-1}$, ${\dot{\varepsilon}}_{4} = 2\times {10}^{-3} \, {\text{s}}^{-1}$). Black arrows indicate major stress drops associated to plastic events.

Figure 3 shows two stress–strain curves for InSb micro-pillars deformed with the strain rate jump protocol and for two diameters (2 µm and 5 µm). Stress drops are visible on the curves within the plastic domain and are the footprint of plastic events. Some of them, corresponding to large stress drops (see black arrows), have a significant impact on the capacity to properly analyze the impact of strain rate jump: in such case, the analysis was not performed and more tests were performed to have sufficient robust data. It is interesting to note that more stress drops are present on curves associated to 2 µm pillars. The latter curves suffer also from higher noise due to the reduced applied force for the same given load cell resolution.

**Figure 4:**
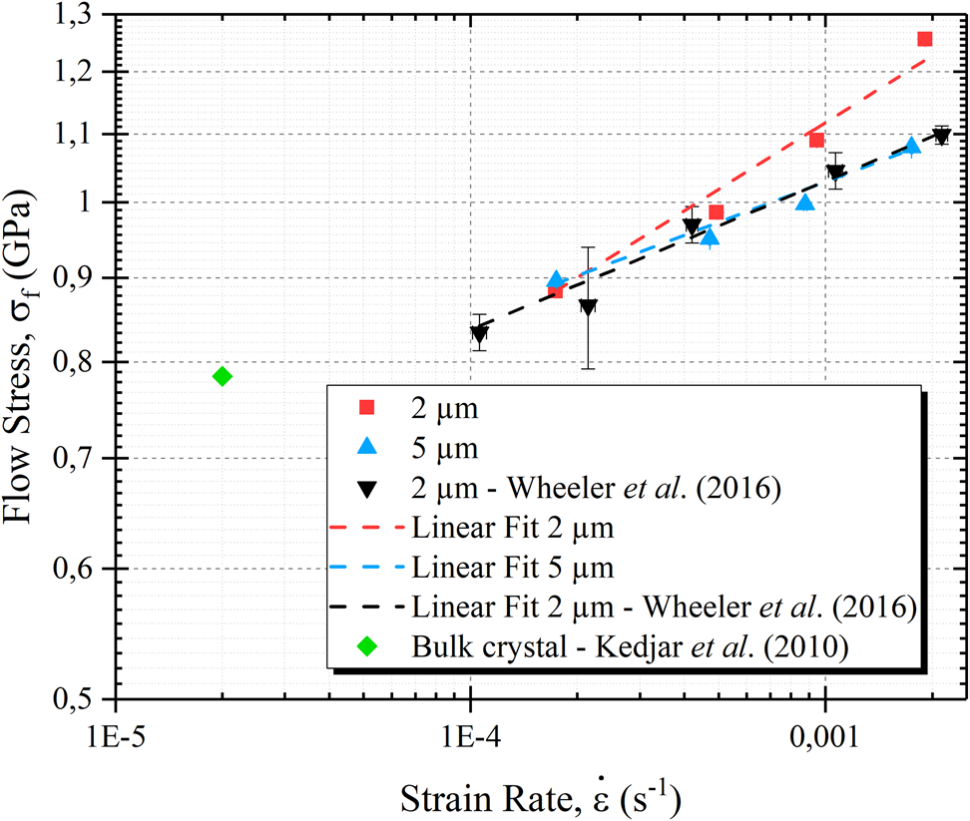
Flow stress with respect to the strain rate for 2 µm, 5 µm micro-pillars and compared to Wheeler et al. results^20^.

Figure 4 presents a log–log plot of the evolution of the flow stress with respect to the strain rate for 5 µm and 2 µm micro-pillars, as measured in the present study, together with recent results from Wheeler et al*.*^20^. Strain rate sensitivity ${\text{m}}$ is deduced from these data by fitting the curves and extracting the slopes, as defined by Eq. 1 (in Methods section). Sensitivity coefficient values are presented in Table 1: *m*(2 µm) = 0.124 and *m*(5 µm) = 0.081. Wheeler et al*.* value is also added for comparison to Table 1.

**Table 1:** Sensitivity *m* and activation volume for 2 µm and 5 µm micro-pillars compared to Wheeler et al. data.

Data set	Sensitivity $m$	${V}_{app}$(${b}^{3}$)—Partial dislocation	${V}_{app}$(${b}^{3}$)—Perfect dislocation
InSb – 2 µm	0.124 ± *0.001*	3.0 ± *0.4*	0.58 ± *0.07*
InSb – 5 µm	0.081 ± *0.005*	4.7 ± *0.5*	0.9 ± *0.1*
Wheeler et al.^20^	0.090 ± *0.005*	4.4 ± *0.5*	–

Activation volumes ${\text{V}}_{\text{app}}$ are calculated using Eq. 2 and sensitivity $m$ values, considering two types of dislocations: perfect dislocation $\text{a}/ 2\langle {110}\rangle$ from $\left\{{111}\right\}$ slip system ($\Vert \overrightarrow{\text{b}}\Vert = 4.582$ Å) and Shockley partial dislocation ${\text{a}}/ 6\langle {112}\rangle$ ($\Vert \overrightarrow{\text{b}}\Vert = 2.645$ Å) from $\left\{{111}\right\}$ slip system. Assuming perfect dislocations leads to low activation volumes: ${\text{V}}_{\text{app}}\left(2\,\upmu\text{m}\right){ = 0.58}\,{\text{b}}^{3}$ and ${\text{V}}_{\text{app}}\left(5\,\upmu\text{m}\right){ = 0.9}\,{\text{b}}^{3}$. In the case of partial dislocation assumption, activation volumes are higher with a value between 3 and 5 b^3^, respectively for micro-pillars with diameter of 2 µm and 5 µm. Apparent activation volumes are summarized in Table 1.

**Figure 5:**
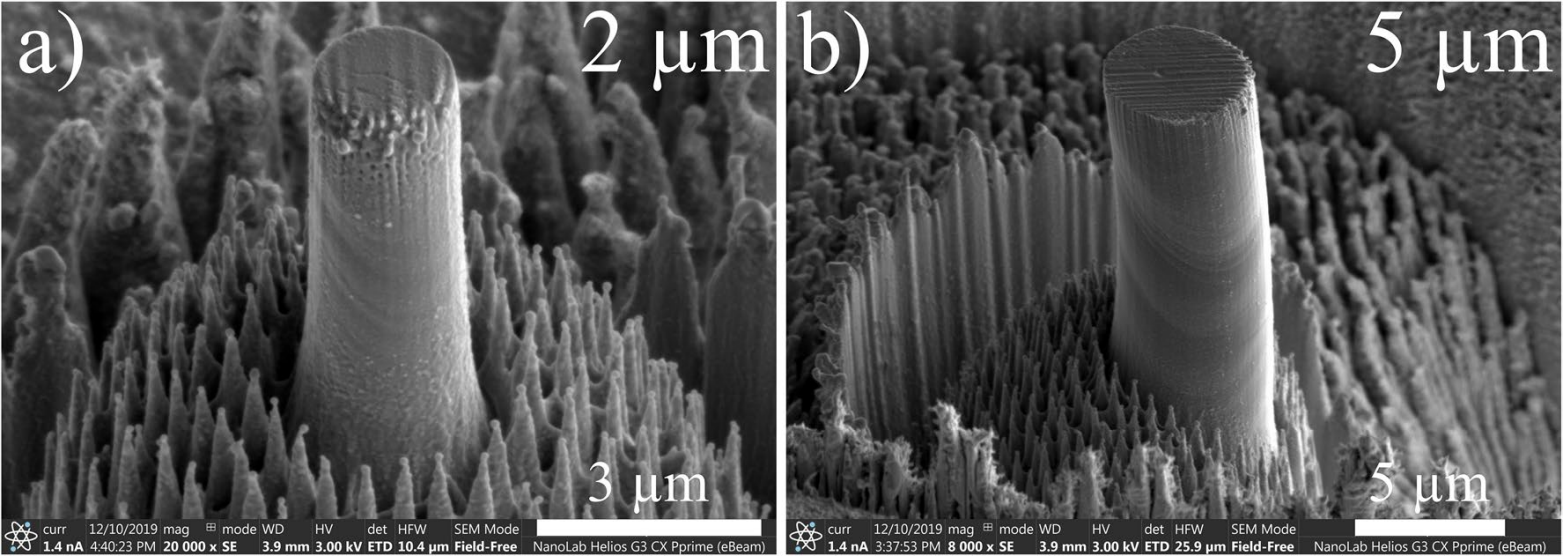
InSb micro-pillars with a diameter of 2 µm and 5 µm after deformation and taken with a tilt of 52°. The sides presented here are the same for both SEM images with respect to the sample orientation.

**Figure 6:**
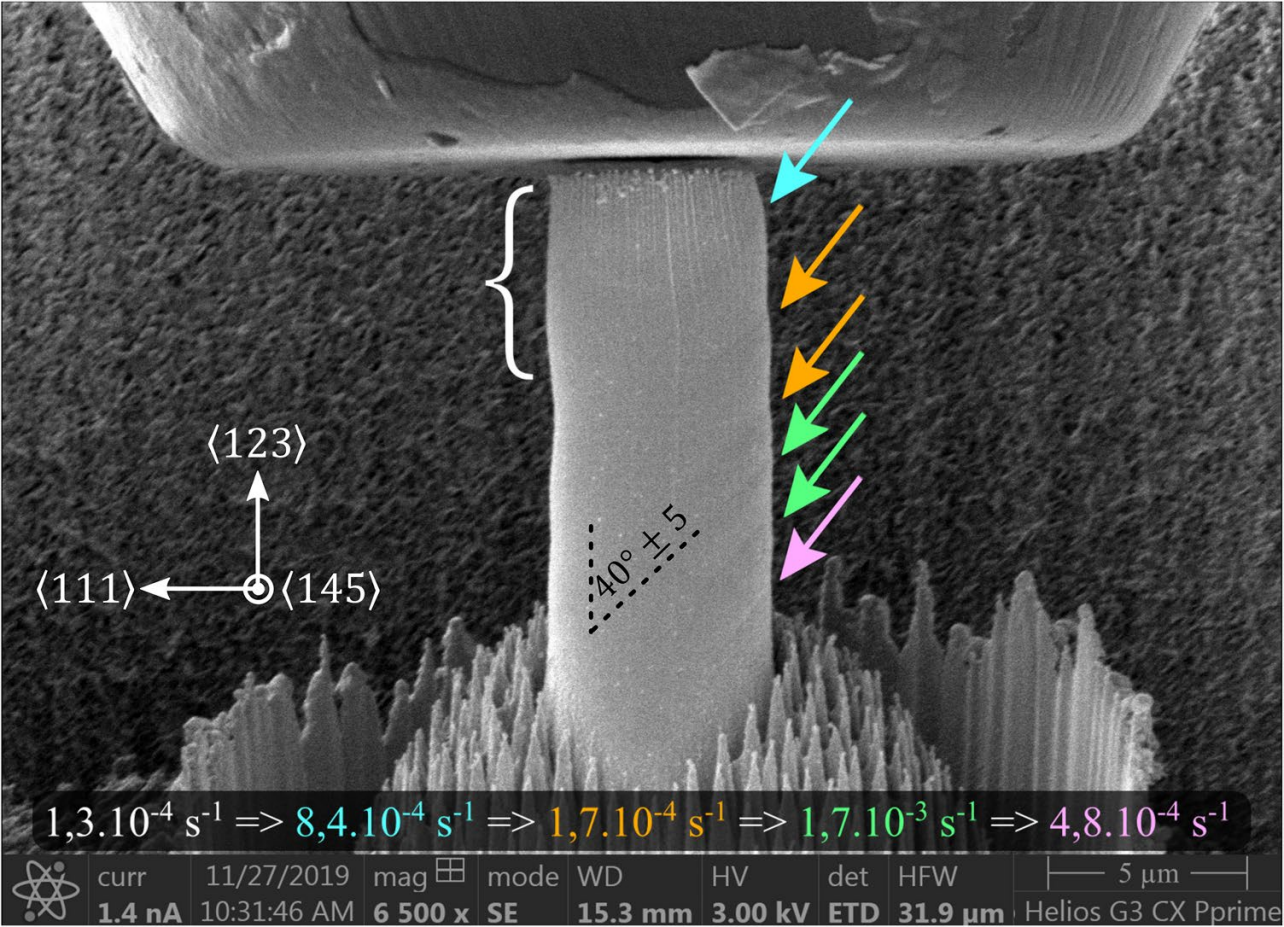
SEM snapshot from the video in supplement material showing an InSb micro-pillar of 5 µm in diameter after the deformation by the strain rate jumps: the white bracket delimits the swelling on top of the pillar, the arrows locate the slip traces at the surface and the different colors indicate in which strain rate regime slip bands appear.

Figure 5 shows SEM images of one 2 µm micro-pillar and one 5 µm micro-pillar after micro-compression (same micro-pillars as presented in Fig. 1). The following observations can be made:

- A homogenous swelling appears at the top of deformed micro-pillars. This swelling can be found on most of InSb micro-pillar after deformation, but it is only visible when observing perpendicular to the primary slip direction.
- Groups of slip traces are visible at the surface for both sizes: they extend in a direction parallel to $\langle {111}\rangle$, with an angle of 40° ± 5 compared to compression axis (relevant crystallographic directions and angle are given in Fig. 6). However, individual slip steps at surface cannot be resolved at SEM scale.
- Groups of slip bands are present all along the pillar height, forming a macroscopic deformation band.

By analyzing deformation videos, the following deformation scenario can be described, for all pillars:

- The top of the pillar deforms first, leading to the previously mentioned swelling.
- Then slip traces appear lower in the body of the pillar, mostly from top down to bottom forming, in the end, a deformation band.

This scenario is illustrated by Fig. 6 which present a 5 µm micro-pillar after a series of strain rate jumps: the white bracket delimits the swelling on top of the micro-pillar and arrows locate the slip traces visible at the surface. The different colors used for the arrows are related to the strain rate regime during which these slip bands appeared. The entire video is available on supplement material and illustrates the apparition and the propagation of slip band over time for InSb micro-pillars with diameter of 5 µm and at 20 °C.

**Figure 7:**
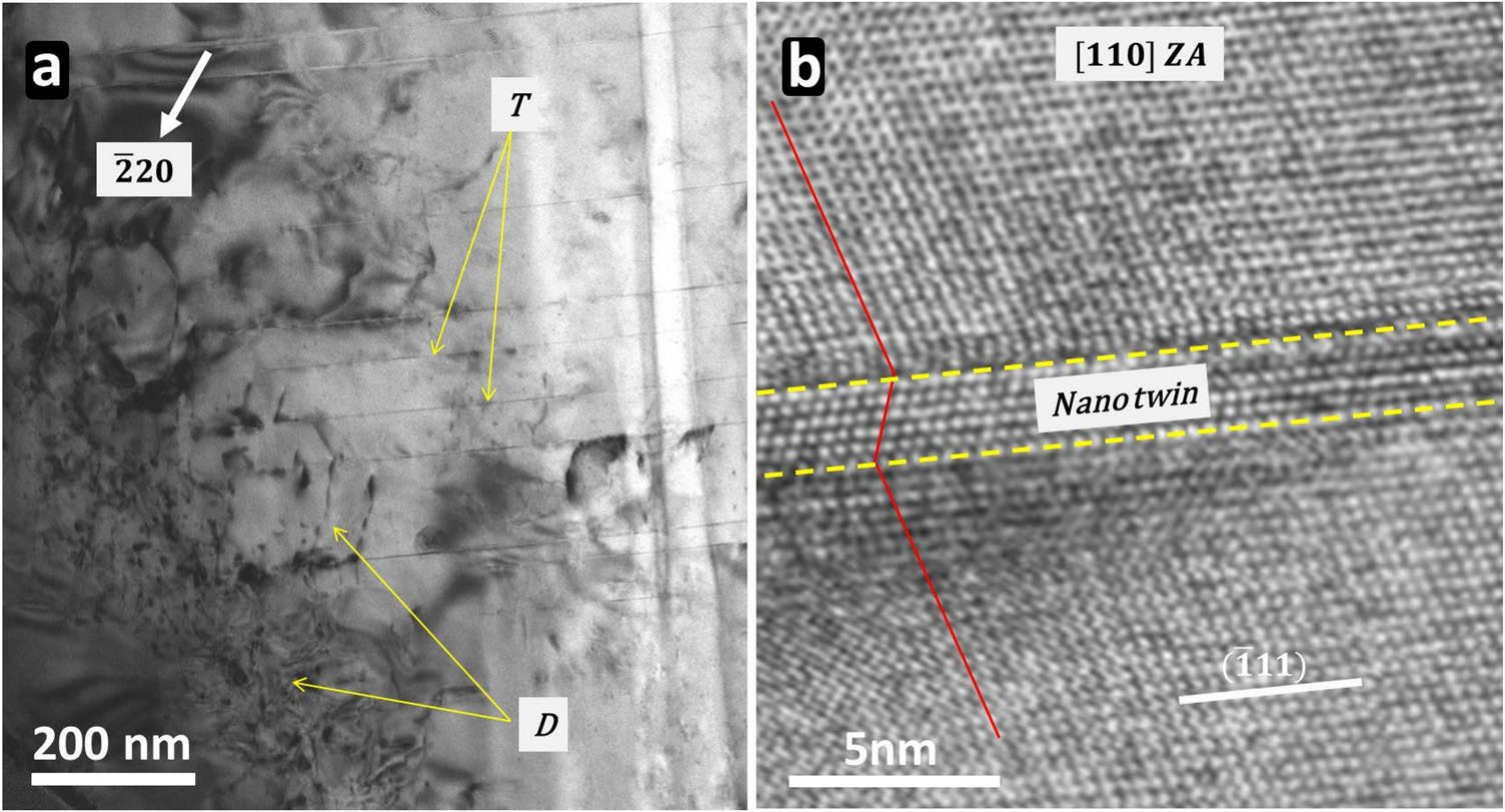
Bright field of a TEM transversal lamella of an InSb micro-pillar deformed at 20 °C in (**a**) and an HRTEM image revealing a nano-twin inside this lamella in (**b**).

Transmission electron microscopy (TEM) has been performed to determine the microstructure of InSb deformed micro-pillars. Figure 7a presents a BF TEM image taken in two-beam configuration of a transversal thin foil extracted from a 5 µm InSb deformed micro-pillar. This thin foil is situated at the transition between the swelling and the middle of the micro-pillar. A high density of dislocations is observed, labeled as *D*. Long and thin straight defects are also visible on the microstructure (labelled *T*) and are parallel to each other. HRTEM analysis has been made (Fig. 7b) and reveal that *T* defects are nano twins, a few atomic layers thick.

## Discussions

Micro-compression at 20 °C induces significant plastic deformation of InSb micro-pillars with a few micrometers in size and this with limited formation of cracks. Cracks only appear when the whole pillar body is plastically deformed (i.e. the deformation band reaching the bottom of the pillar). This result is in agreement with studies reported in Thilly et al*.*^19^ and Wheeler et al.^20^, where plasticity could not be reached in InSb micro-pillars with size larger than 15 µm. A comparison with the bulk is also made in Fig. 4 which presents the flow stress as a function of the strain rate. It appears that the stress is higher for micro-pillars. This difference can be attributed to the density of defect which is statistically much smaller in micro-pillars compared to a bulk sample.

During the plasticity regime, large number of stress drops are observed on the stress–strain curves, with variable magnitude (Figs. 1 and 2). Such events can naturally be attributed to avalanches of dislocations which are known to produce such features in defect-free crystals deformed under displacement control mode. It is interesting to note that some strain hardening is present in most of the stress–strain curves, and it seems to increase with the strain rate as shown in Fig. 2.

By combining the in-situ SEM videos and post-mortem SEM analysis, the following remarks can be made with respect to the deformation sequence of InSb micro-pillars:

- The top part of the deformed micro-pillars exhibits a complex deformation volume (the “swelling”) that can be related to several technical considerations. First, the slight misalignment between the flat punch and the top surface of the micro-pillar (smaller than 2°) leads to a stress concentration at one edge of the top surface; this phenomenon is further reinforced by the taper generated by the milling method, leading to a slightly smaller cross-section in the top region of the micro-pillar. Second, contact between the flat punch and the top surface of the micro-pillar leads to friction inherently due to the compressive nature of the mechanical test. All these issues induce a modification of the stress tensor both in magnitude and in its components in the top part of the micro-pillars: the first consequence is the loss of uniaxial nature of the stress that may lead to the activation of several slip systems, despite single-slip orientation of the crystal. Consequently, a higher density of defects is predictable in the top volume of micro-pillars: this is in line with the observed homogenous swelling as evidenced by the white bracket in Fig. 5. The interaction of several slip systems in this region may also explain the presence of different types of dislocations. It is interesting to note that after the plastic deformation of the top region, the micro-pillar top surface is in full contact with compression flat punch.
- The majority of the micro-pillars deformation profile is characterized by the appearance of successive parallel slip bands along the micro-pillar body as the compression progresses. This is well illustrated by the video available in the supplementary materials and by Fig. 6 which highlights the order of appearance of the plastic footprints. This deformation mode is explained by the fact that, after plastic deformation of the top region leading to full contact between micro-pillar top surface and flat punch, the stress becomes uniaxial which leads to the activation of the expected primary slip system, in the form of parallel slip bands. Noteworthy, the orientation of the slip traces agrees with the primary slip system (experimental angle of 40° ± 5 to be compared with a theoretical value of 38°).

In summary, the deformed top region is associated to the onset of plasticity in the micro-pillars and coincides with the elastic–plastic transition of the stress–strain curve. The next step is the formation of slip bands that progresses from top to bottom, thus forming an extended deformation band: this step coincides with the extended plastic regime seen in the stress–strain curves.

The determination of the strain rate sensitivity *m* is performed by strain rate jumps during the plastic regime seen in the stress–strain curves. As such, the outcomes of this technique should be representative of the deformation mechanisms activated during this regime, i.e., the progress of the slip bands as described above. The obtained *m* values are 0.124 ± 0.001 for 2 µm micro-pillars and 0.081 ± 0.005 for the 5 µm micro-pillars. By comparing with Wheeler et al*.* results (*m* = 0.09 ± 0.005), the order of magnitude is in good agreement. It must be noted that Wheeler et al*.* only deformed 2 µm InSb micro-pillars. The slightly different *m* values obtained here for 2 µm and 5 µm micro-pillars might be partly explained by the several plasticity events appearing preferentially on the 2 µm curves which prevent the best fit of the jumps and by the higher noise due to the load cell resolution.

Strain rate sensitivity is used to calculate the apparent activation volume ${\text{V}}_{\text{app}}$. Very low values, i.e. 0.58 b^3^ for 2 µm and 0.9 b^3^ for 5 µm, are found when assuming that plasticity is mediated by perfect dislocations slip: such values are too low to be compatible with the motion of dislocations^21,22^. Indeed, the activation volume is related to the local volume impacted by the motion of defects in the crystal lattice and a value below 1 b^3^ is generally associated to diffusion mechanisms, as also concluded by Wheeler et al.^20^. The other hypothesis is that plasticity is mediated by partial dislocation slip: such case yields an apparent activation volume of 3–5 b^3^. This range is consistent with the value found by Wheeler et al*.* under the same assumption. Also, the small discrepancy between activation volume values obtained from 2 and 5 µm diameter micro-pillars might be explained by the similar discrepancy found between strain-rate sensitivity values for these micro-pillar dimensions. Overall, activation volume values point towards the slip of partial dislocations, as the main plasticity mechanism associated to the formation of slip bands along the micro-pillar body.

After these considerations, TEM analysis allowed to check the relevance of the proposed plasticity mechanisms. In the present paper, the focus is made on the analysis of the transversal TEM foil presented in Fig. 7 that is representative of all foils that have been studied. It should be recalled that the observed region is at the transition between the “swelling” and the top part of the deformation bands composed of parallel slip bands. The top part of the micro-pillars is governed by a complex plastic regime which is characterized by the activation of multiple slip systems inducing the nucleation of a lot of dislocations. These dislocations interact with each other leading to a high density of defects labelled *D* in Fig. 7.

Transversal thin foil reveals the presence of straight defects which are actually parallel individual nano-twins. They are created by the nucleation and motion of leading partial dislocations in successive atomic layers which is consistent with the observation of deformation bands at the surface (Fig. 5) and the activation volume values in Table 1. The activation of a large quantity of parallel slip planes requires a lot of dislocations sources. As mentioned above, FIB milling creates a high quantity of defects at the very surface of micro-pillars during the fabrication process that are susceptible to be nucleation sites for dislocations. Indeed, Guénolé et al*.*^23^ showed that the interface between an amorphous layer and the crystalline region of Si sample contains native atomic defects acting as dislocation sources.

The observation of nano-twins confirms that the deformation of the micro-pillar body proceeds by the nucleation and glide of Shockley partial dislocations, as already suggested by the activation volume values. It should be recalled that the presence of partial dislocations at 20 °C was already observed in previous work in bulk crystal^6^ and InSb micro-pillars^19^. Moreover, the results from Kumar et al.^9^ on the GSFE determination in InSb, demonstrated that dissociation is only possible in the GS. The new result here is the presence of nano-twins: their formation can only be the result from a “collaborative” mechanism where a new partial dislocation is preferably nucleated in the adjacent plane to a previous partial dislocation glide plane. Such collaborative mechanism is proposed to be specific to the micro-pillar case, as a combined consequence of the presence of large surface-to-volume ratio (and associated high density of nucleation sites—see above) and high stress (strong driving force for nucleation).

In general, properties of materials can be modified by the presence of nano-twins^24^. This is particularly the case of semiconductors, which optical properties are impacted^25–27^. Nano-twins can also be used to enhance the thermoelectric properties of semiconductors as suggested by Zhou et al*.*^28^, who found that incorporating nano-twins in Si nanocrystalline heterostructures increases the electrical conductivity and decreases the thermal conductivity. In the case of InSb, nano-twin boundaries influence the electron mobility^29–31^. Our results suggest that the deformation of small-scale electronic devices made of InSb induces nano-twins which, in turn, may modify the devices opto-electronic properties.

In summary, micro-pillars of InSb of 2 and 5 µm were fabricated by FIB milling to study their plasticity mechanism at small-scale. In situ deformation was performed with constant strain rate and led to plastic activity characterized by stress drops, strain hardening and the formation of slip traces. Strain rate jumps were successfully performed and sensitivity ${\text{m}}$ was extracted from the micro-compression tests. Activation volumes ${\text{V}}_{\text{app}}$ were calculated considering two assumptions: perfect dislocations in the ${\text{a}}/ 2\langle {110}\rangle \left\{{111}\right\}$ slip system and partial dislocations in the ${\text{a}}/ 6\langle {112}\rangle \left\{{111}\right\}$ slip system. Partial dislocation slip is the only one found to be sensible with an apparent activation volume between 3 and 5 b^3^. Sensitivity and activation volume values are also in good agreement with previous work^20^.

SEM analysis highlighted two different regimes in deformation of InSb micro-pillars: the top part is deformed in a complex manner because of mechanical constraints (misalignment, stress concentration, friction at the contact…) which modify the stress tensor; the rest of the micro-pillars’ body is deformed in an uniaxial manner where parallel slip traces emerge at surface and form deformation bands. TEM thin foils of deformed InSb micro-pillars revealed the presence of nano-twins which constitutes a new deformation mechanism of InSb micro-pillars below 5 µm and at 20 °C.

The formation of nano-twins can be explained by the preferential nucleation of partial dislocations in successive slip planes, a mechanism favored by features that are specific to small-scale InSb sample, or “size effect”: higher density of nucleation sites at surface and enhanced stress compared to bulk sample. In view of the potential impact of nano-twins on InSb opto-electronic properties, it is thus crucial to account for this deformation mechanism in the design of small-scale InSb devices in a context of continued miniaturization.

## Methods

InSb single crystal used in this study is oriented along $\langle {123}\rangle$ direction to promote the activation of only one slip system: ${\text{a}}/ 2\langle {110}\rangle \left\{{111}\right\}$ with a Schmid factor of 0.47. Samples have been chemically polished with a hydrofluoric acid solution^5,6^ to obtain a sample with low defects density.

A FIB Helios NanoLab G3 CX (from ThermoFischer Scientific) with a gallium source was used to mill cylindrical InSb micro-pillars via the annular method (see below), as it strongly reduces the impact of the ion beam on the crystal structure. Micro-pillars were all fabricated at the edge of the sample giving access to the lateral view and are all sitting on a pedestal to ensure a good stability during mechanical tests. Two platinum layers were successively deposited to protect the future micro-pillar during the ion milling: the first one with the electron beam and the second one with the ion beam. A first annular milling step was performed with a high ion beam current of 9.1 nA to create a large annular crater. Successive annular milling steps with decreasing dimensions and lower current were performed to obtain each micro-pillar, sitting on its pedestal. The final “polishing” step was performed with a low current of 80 pA to reduce damage. Ion beam voltage was set constant at 30 kV. It must be noted that a chemical reaction occurs between gallium and indium at 15 °C: this phenomenon strongly disturbs the fabrication of micro-pillars by increasing the milling rate and potential damage onto the lattice. This reaction is also mentioned in a few studies^19,32^ and leads to a specific surface microstructure characterized by significant redeposition. To limit this effect, dwell time of the ion beam must be set to its minimum (25 ns): this leads to a reduced surface exposure to the ion beam, while maintaining milling. At the end of the fabrication sequence, the adjustment of the pillar height is performed by tilting the sample perpendicular to the ion beam: this step enables to control the pillar aspect ratio (height over diameter, defined at a value of 3, for mechanical stability), to remove the platinum top layers and to flatten the top surface of micro-pillars. This step is done at 16 kV and 50 pA to further limit induced damage. Two pillar diameters are considered in this study: 2 µm and 5 µm. The fabrication protocol induces a difference in diameter between the top and the base of the micro-pillars. In other words, micro-pillars are not perfectly cylindrical but exhibit a slightly conic shape which can be characterized by a vertical angle along the micro-pillars axis and called taper. Here, the taper angle is always smaller than 3.5°.

Extraction of TEM thin foil was made by FIB milling on deformed InSb micro-pillars using a modified Lift-Out method. Indeed, conventional Lift-Out method^32,33^ was initially developed for the extraction from bulk sample but special considerations are needed here for micro-pillars such as the orientation of the final thin foil (that depends on the cutting orientation), the technique to glue the foil onto the TEM grid and the thinning parameters that must take into account the previously mentioned chemical reaction. Different thin foil orientations were used but the present study only focuses on the transversal thin foils, which are extracted at 90° from the micro-pillar (compression) axis. The crystalline quality of the extracted thin foils was checked by extracting foils from non-deformed micro-pillars. An amorphous layer of about 10 nm in thickness was observed by HRTEM at the micro-pillar surface and the transition between this amorphous layer and the crystalline region was found to be very sharp. This sharp transition was also seen by Thilly et al*.*^19^. Despite the chemical reaction, FIB thinning can therefore be controlled to induce a very low density of structural defects and allowing the proper study of the deformed microstructure.

The TEM examinations were performed using a ThermoFisher Scientific Talos F200S G2 equipped with a double tilt holder and operated at an acceleration voltage of 200 kV. The point resolution of this microscope in TEM mode is 0.12 nm. Conventional TEM imaging was performed in Bright Field (BF) mode using two-beam diffraction condition. High Resolution (HRTEM) imaging was performed along $\left[{110}\right]$ Zone Axis (ZA) which is near the thin foil normal direction.

Micro-compressions were performed in situ inside the FIB chamber using a FIB/SEM-compatible nano-indenter from ALEMNIS^34^. This configuration allows recording videos of mechanical tests by taking SEM images and videos. The mechanical tests were made in displacement control with a flat punch of 20 µm in diameter (from SYNTON – MDP) and at room temperature. The load cell has a root mean square (RMS) noise of 4 µN at 200 Hz bandwidth and the displacement unit has a RMS noise of 2 nm at 200 Hz bandwidth^34^. Four strain rates were chosen: ${\dot{\varepsilon}}_{1} = 1.5\times {10}^{-4} \, {\text{s}}^{-1}$, ${\dot{\varepsilon}}_{2} = 5\times {10}^{-4} \, {\text{s}}^{-1}$, ${\dot{\varepsilon}}_{3} = {10}^{-3} \, {\text{s}}^{-1}$ and ${\dot{\varepsilon}}_{4} = 2\times {10}^{-3} \, {\text{s}}^{-1}$. Several corrections are applied to obtain the true stress-true strain curves: (1) the contribution from the nano-indenter compliance ^35^ (which is automatically removed by the data analysis software), (2) the contribution from the elastic deformation of the pedestal (“sink-in” phenomenon)^36,37^ and (3) the load cell and thermal drifts estimated and corrected by the software. Micro-compressions with constant strain rate were performed to probe the plastic response of micro-pillars and to estimate the strain hardening at each strain rate value and each micro-pillar size. Strain rate jumps were also performed to calculate the strain rate sensitivity $\it {\text{m}}$ determined by the following formula, with ${\sigma}_{\text{f}}$ the flow stress and $\dot{\varepsilon }$ the strain rate^21^:

1 $${m} = \frac{\partial{\text{log}}({\sigma}_{\text{f}})}{{\partial}{\text{log}}(\dot{\varepsilon})}$$

The flow stress that corresponds to the stress needed to propagate the plasticity is calculated from the strain rate jumps. Each jump is analyzed independently: each corresponding stress–strain curve portion is fitted by a power law equation to extract the flow stress as the asymptotic value of the fit. Power law equations are adjusted using the least squares method. In the case that a strain rate jump effect includes a strain hardening contribution (evidenced from constant strain test), a correction is applied using a linear regression to remove the hardening contribution. Strain rate sensitivity $\it {m}$ was also used to calculate the apparent activation volume ${\text{V}}_{\text{app}}$ that corresponds to the number of atoms involved during the motion of a dislocation in the lattice (with or without pre-existing defects). It depends on the temperature ${\text{T}}$ (in Kelvin), the flow stress ${\sigma}_{\text{f}}$ and the sensitivity $\it {m}$ as shown by the following formula:

2 $$\it {\text{V}}_{\text{app}}=\frac{\sqrt{3}{{\text{k}}}_{\text{b}}{\text{T}}}{{\sigma}_{\text{f}}\times {m}}$$

Apparent activation volume is usually expressed in b^3^ (where *b* is the magnitude of the Burgers vector) and depends on the considered dislocation nature. A small value of activation volume means that a small number of atoms are involved in the motion of a dislocation (e.g., through the dislocation core).

## Supplementary Information

Supplementary Video 1.
